# ARC6-mediated Z ring-like structure formation of prokaryote-descended chloroplast FtsZ in *Escherichia coli*

**DOI:** 10.1038/s41598-017-03698-6

**Published:** 2017-06-14

**Authors:** Hiroki Irieda, Daisuke Shiomi

**Affiliations:** 1 Department of Life Science, College of Science, Rikkyo University, Tokyo, 171-8501 Japan; 2 Present Address: Academic Assembly, Institute of Agriculture, Shinshu University, 8304 Minamiminowa, Kami-Ina, Nagano 399-4598 Japan

## Abstract

Plant chloroplasts proliferate through binary fission, and the stromal-side molecules that are involved in chloroplast division are bacterial derivatives. As in bacteria, the prokaryotic tubulin homolog FtsZ assembles into a ring-like structure (Z ring) at mid-chloroplast, and this process is followed by constriction. However, the properties of chloroplast FtsZs remain unclarified. Here, we employed *Escherichia coli* as a novel heterologous system for expressing chloroplast FtsZs and their regulatory components. Fluorescently labelled Arabidopsis FtsZ2 efficiently assembled into long filaments in *E. coli* cells, and artificial membrane tethering conferred FtsZ2 filaments with the ability to form Z ring-like structures resembling the bacterial Z ring. A negative regulator of chloroplast FtsZ assembly, ARC3, retained its inhibitory effects on FtsZ2 filamentation and Z ring-like structure formation in *E. coli* cells. Thus, we provide a novel heterologous system by using bacterial cells to study the regulation of the chloroplast divisome. Furthermore, we demonstrated that the FtsZ2-interacting protein ARC6, which is a potential candidate for Z ring tethering to the chloroplast inner envelope membrane, genuinely targeted FtsZ2 to the membrane components and supported its morphological shift from linear filaments to Z ring-like structures in a manner dependent on the C-terminal ARC6-interacting domain of FtsZ2.

## Introduction

Plant chloroplasts are descended from ancient photosynthetic prokaryotes through endosymbiosis, and chloroplasts proliferate by growth and division, similar to bacteria^1,2^. The chloroplast division machinery is a hybrid structure that is composed of both bacterial and host-derived elements, which localize on the inner (stromal) and outer (cytosolic) sides, respectively^3^. In bacterial cells, a tubulin-like GTPase, FtsZ, is a key component that assembles into a ring-like structure (Z ring) at mid-cell and that contributes to cell constriction. However, whether FtsZ actually generates a constriction force in the cell or functions as a mobile scaffold for peptidoglycan synthesis is controversial^4–8^. As in bacteria, FtsZ homologs in chloroplasts assemble into a Z ring in the stromal side immediately beneath the inner envelope of the chloroplast membrane at the future site of division (mid-chloroplast), following which other division-related components are recruited^3,9–11^. Over the course of evolution, many chloroplast genes, including *FtsZ* and other division-related genes, were transferred to the nuclear genome, but the encoded proteins are transported back to chloroplast because of their N-terminal chloroplast transit peptide (cTP)^9,10,12,13^. Chloroplasts contain two FtsZ homologs, FtsZ1 and FtsZ2 (the latter of which is encoded by two functionally redundant genes, *AtFtsZ2-1* and *AtFtsZ2-2*, in *Arabidopsis thaliana*)^14,15^; AtFtsZ1 and AtFtsZ2 form heteropolymers both *in vitro* and in yeast cells^16–19^, play functionally distinct roles, and are essential for chloroplast division^14,15,18^.

Chloroplast FtsZ proteins lacking the cTP share high sequence identity and similarity with bacterial FtsZs, especially in their GTPase core domain (see Supplementary Fig. S1). Moreover, the C-terminal domain critical for the interaction of FtsZ with FtsA/ZipA, which anchor FtsZ to the membrane in bacteria^20,21^, is partially conserved in chloroplast FtsZ2 (see Supplementary Fig. S1), although FtsA/ZipA homologs are not present in plants. FtsZ1 lacks this interaction sequence in its C-terminus, implying that *FtsZ1* emerged from *FtsZ2* through gene duplication during evolution^22,23^. With regard to whether chloroplast FtsZs also generate a constriction force like bacterial FtsZ^4^, a recent study reported that fluorescently tagged AtFtsZ1 and/or forcibly membrane-targeted AtFtsZ2 expressed in cells of the yeast *Pichia pastoris* form ring-shaped filaments, and the rings, including the AtFtsZ2 homopolymer or AtFtsZ1/AtFtsZ2 heteropolymer, were found to contract^19^. In contrast, previous research on the division machinery of chloroplasts isolated from the red alga *Cyanidioschyzon merolae* have suggested that the motive force of chloroplast constriction was not provided by the internal Z ring, but rather by the external, dynamin-like DRP5B ring^19^. Thus, the mechanism by which FtsZs function in chloroplast division remains obscure as compared with what is known regarding their bacterial counterparts.

*In planta* analysis of chloroplast FtsZs, wherein several components of the division system act together, is critical to comprehensively evaluate the role of FtsZ during chloroplast division. *In vitro* and heterologous expression systems can also be used to enhance our understanding of the inherent properties of chloroplast FtsZs. To date, the fission yeast *Schizosaccharomyces pombe* and the methylotrophic yeast *P. pastoris* have been successfully used as heterologous expression systems for chloroplast division-related components, including AtFtsZ1 and AtFtsZ2^18,19,24,25^. In *S*. *pombe* cells, linear and ring-shaped filaments of AtFtsZ1 and/or AtFtsZ2 in the free-floating state in the cytosol were observed^18,25^. The dominance of AtFtsZ2 over AtFtsZ1 in filament morphology and the faster turnover of AtFtsZ1 compared with AtFtsZ2 have led to a proposal that AtFtsZ2 forms the filament backbone structure and AtFtsZ1 facilitates filament remodeling, but the typical Z ring formation of AtFtsZs alongside membrane structures such as the bacterial plasma membrane or the chloroplast inner envelope membrane has not yet been reported in the fission yeast system^18,25^. However, a recent study conducted using *P. pastoris* cells revealed that AtFtsZ2 tagged with the membrane-targeting sequence (MTS) from *Escherichia coli* MinD protein assembled into a contractible ring whether or not AtFtsZ1 was present^19^. This AtFtsZ2 ring was shown by fractionation analysis to be attached to the cell membrane, although it remained unclear whether this genuinely reflected the typical Z ring condition in which the filament is tethered perpendicularly to the membrane plane.

Here, we established a novel heterologous expression system by using the model bacterium *E. coli* in order to monitor the assembly and dynamics of chloroplast FtsZ expressed together with or without other regulatory proteins such as ARC3 and ARC6. As already mentioned, chloroplasts originated from free-living bacteria, and the chloroplast Z ring is formed in the space inside the inner envelope membrane, the stroma, which is topologically equivalent to the bacterial cytosol. Therefore, we sought to develop a feasible system for the heterologous reconstitution of chloroplast Z ring-associated machinery by using *E. coli* cells to evaluate the function of chloroplast FtsZ and its related components.

## Results

### Chloroplast FtsZ2 assembles into filaments in *E. coli* in a manner partially dependent on its N-terminus

**Figure 1:**
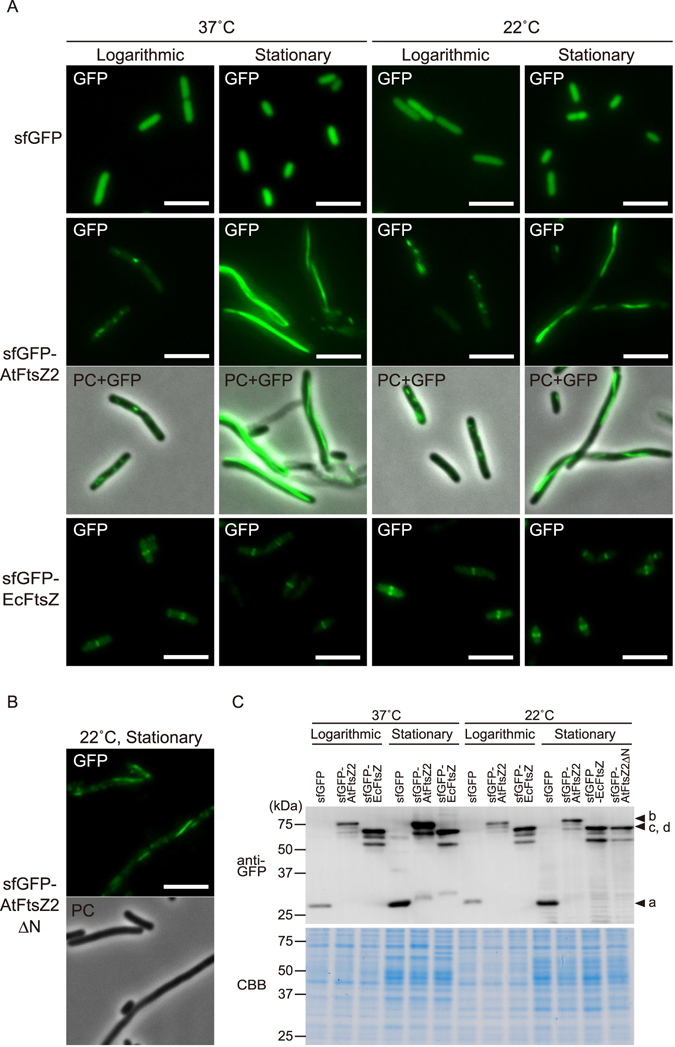
Chloroplast FtsZ2 assembles into filaments in *Escherichia coli* in a manner partially dependent on its N-terminus. (**A**) Fluorescence images of sfGFP-tagged FtsZ proteins from *Arabidopsis thaliana* (AtFtsZ2) and *E. coli* (EcFtsZ) expressed in *E. coli* wild-type strain WM1074 under various conditions. Scale bar: 5 μm. (**B**) Filament morphology of sfGFP-AtFtsZ2 lacking the extra N-terminal region, in the stationary phase at 22 °C. Scale bar: 5 μm. (**C**) Anti-GFP immunoblotting of each FtsZ protein under various conditions. Arrowheads indicate the full-length band of sfGFP (a), sfGFP-AtFtsZ2 (b), sfGFP-EcFtsZ (c) and sfGFP-AtFtsZ2ΔN (d). Coomassie stain was used as a loading control.

Only one report to date has shown the localization pattern of AtFtsZ2 expressed in *E. coli* (in an *ftsZ*-depleted strain); in these bacterial cells, AtFtsZ2 fused with green fluorescent protein (GFP) at the C-terminus (AtFtsZ2-GFP) did not form Z rings but assembled into long filaments and large spots in the bacterial cytoplasm^18^. One possible explanation for the formation of the large (fluorescent) spots of AtFtsZ2-GFP is that inappropriate conditions used for heterologous expression of chloroplast FtsZ in *E. coli* might have led to aberrant aggregation of the molecule. To test whether AtFtsZ2 can form Z rings in *E. coli* cells under certain conditions, we first fused superfolder GFP (sfGFP) to the N-terminus of chloroplast transit peptide (cTP)-lacking AtFtsZ2-1 (sfGFP-AtFtsZ2) and expressed it in the *E. coli* wild-type strain WM1074 at the optimal temperature for either *E. coli* (37 °C) or *A. thaliana* (22 °C) (Fig. 1A). Under the tested conditions, AtFtsZ production resulted in the elongation of *E. coli* cells, implying that AtFtsZ exerts a toxic effect on *E. coli* cell division, presumably by inhibiting the formation or turnover of the Z ring composed of *E. coli* FtsZ (EcFtsZ). In the stationary phase, sfGFP-AtFtsZ2 assembled into long filaments that extended along the long axis of bacterial cells, whereas only small dots and short filaments were observed in cells from logarithmic cultures (Fig. 1A). Notably, upon incubation at 37 °C, lysed cells were frequently observed, which implied that sfGFP-AtFtsZ2 adversely affected cell viability at this temperature. At 37 °C, C-terminally sfGFP-tagged AtFtsZ2-1 (AtFtsZ2-sfGFP) exhibited aberrant aggregation or formation of inclusion body-like patterns at cell poles (see Supplementary Fig. S2A) whereas sfGFP-AtFtsZ2 did not show such aberrant aggregation in *E. coli* cells (Fig. 1A). Moreover, in the previous study, the large spots of AtFtsZ2-GFP were observed at 42 °C^18^. Thus, AtFtsZ2 featuring a C-terminal GFP fusion likely aggregates in *E. coli* cells at high temperatures. Here, the N-terminal sfGFP fusion of EcFtsZ, sfGFP-EcFtsZ, expressed as a control, showed typical mid-cell Z ring formation under all experimental conditions (Fig. 1A). Thus, for subsequent experiments, we used sfGFP-AtFtsZ2 expressed at 22 °C.

Chloroplast FtsZ proteins, including AtFtsZ2, harbor extended N-terminal sequences compared with their bacterial counterparts (see Supplementary Fig. S1). This extra region of AtFtsZ2 can promote polymer bundling and turnover^25^. Therefore, we expressed sfGFP-AtFtsZ2 lacking this extra N-terminal region (AtFtsZ2^residue65-430^) (sfGFP-AtFtsZ2∆N) and examined the morphology of its filaments. Fluorescent imaging and quantitative analysis revealed that sfGFP-AtFtsZ2∆N formed considerably shorter filaments than sfGFP-AtFtsZ2 did (Fig. 1B, see Supplementary Fig. S2B).

Immunoblotting of the FtsZ constructs with an anti-GFP antibody (Fig. 1C) revealed that the full-length product in all cases was detected as the major band and cleaved products were detected as minor bands. Notably, the expression level of sfGFP-AtFtsZ2 was significantly lower than that of sfGFP-EcFtsZ during the stationary phase at 22 °C. In comparison with the FtsZ levels in *A. thaliana* chloroplast and *E. coli*, the number of AtFtsZs molecules was estimated to be 101,200^26^, while that in *E. coli* was approximately 5,000–15,000^27,28^. Given the normal division time of sfGFP-EcFtsZ-expressing cells (Fig. 1A), the observed levels of sfGFP-EcFtsZ were not too high. Additionally, the level of sfGFP-AtFtsZ2 expressed in *E. coli* cells was inevitably lower than that in the chloroplast.

Collectively, these results are consistent with previous work conducted using *S. pombe* cells^18^, and suggest that sfGFP-AtFtsZ2 expressed in *E. coli* cells exhibits its natural molecular properties.

### Membrane-tethered FtsZ2 forms multiple Z ring-like structures

The C-terminal region of EcFtsZ interacts with FtsA, which interacts with the membrane through its C-terminal amphipathic helix^29,30^. Consequently, indirectly membrane-tethered FtsZ forms a Z ring oriented perpendicular to the long cell axis. Accordingly, the results of *in vitro* liposome reconstitution analysis demonstrated that EcFtsZ artificially tethered to the membrane by the C-terminal MTS formed Z rings beneath the membrane and produced liposome constriction^4^. In the case of chloroplasts, the mechanism by which the Z ring is anchored to the inner envelope membrane remains unresolved, but MTS-tagged AtFtsZ2 was reported to form a contractible ring structure in a *P. pastoris* heterologous expression system^19^.

**Figure 2:**
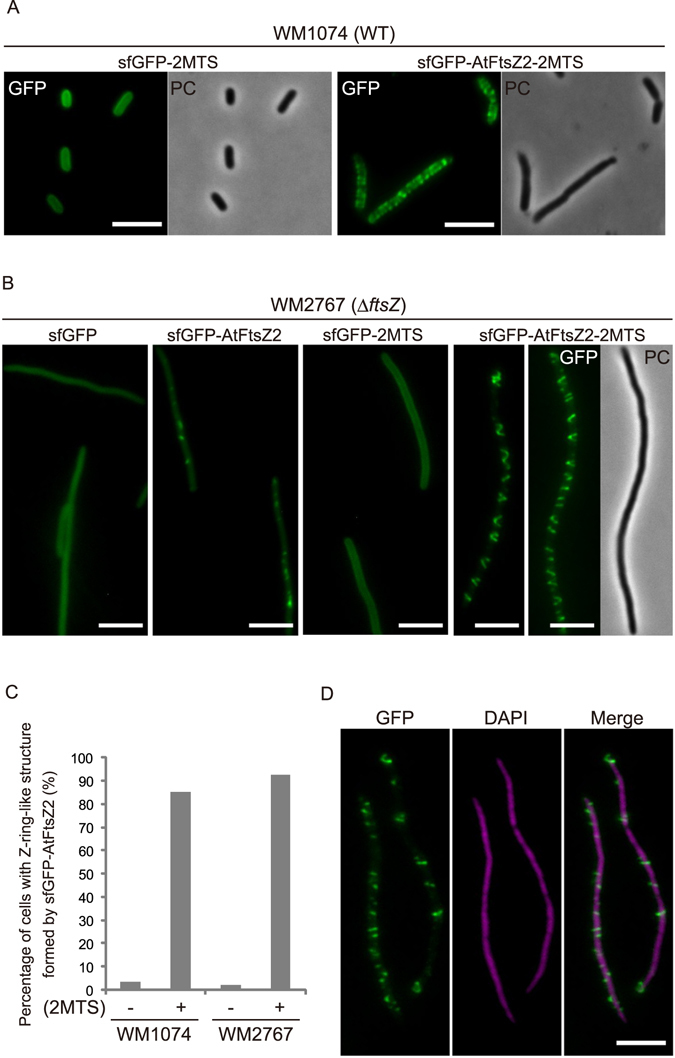
Membrane-tethered FtsZ2 forms multiple Z ring-like structures. (**A**) Fluorescence images of sfGFP-AtFtsZ2 tagged with 2MTS in *E. coli* WM1074 strain. Scale bar: 5 μm. (**B**) Filament morphology of sfGFP-AtFtsZ2 with or without 2MTS in the FtsZ-depleted strain WM2767. Scale bar: 5 μm. (**C**) Quantification of Z ring-like structures formed by sfGFP-AtFtsZ2 with or without 2MTS in the WM1074 and WM2767 strains. The graph shows the percentage of cells containing >1 Z-ring-like structure. The numbers of elongated bacterial cells investigated for the quantification were 212 (WM1074/sfGFP-AtFtsZ2), 123 (WM1074/sfGFP-AtFtsZ2-2MTS), 97 (WM2767/sfGFP-AtFtsZ2), and 41 (WM2767/sfGFP-AtFtsZ2-2MTS). (**D**) Concurrent observation of AtFtsZ2 Z ring-like structures and bacterial chromosomes in the WM2767 strain. Chromosomes were stained with DAPI. Scale bar: 5 μm.

In this context, we added two MTS tags (KGFLKRLFGG) derived from *E. coli* MinD in tandem to the C-terminus of sfGFP-AtFtsZ2 (sfGFP-AtFtsZ2-2MTS), and the fusion protein was produced in *E. coli* wild-type strain WM1074 (Fig. 2A, see Supplementary Fig. S3). This MTS-mediated membrane tethering of AtFtsZ2 in *E. coli* cells resulted in the formation of multiple Z ring-like structures. Next, to eliminate the possibility that endogenous EcFtsZ supported the assembly of AtFtsZ2, the fusion protein was produced in the *ftsZ*-depleted strain WM2767. In this strain, chromosomal *ftsZ* was deleted; instead, *ftsZ* was expressed from a plasmid, and its expression was induced by using sodium salicylate (NaSal). EcFtsZ in this strain was depleted by elimination of the inducer from the medium (see Supplementary Fig. S3). The depletion of endogenous EcFtsZ in this strain caused cell elongation, and these cells were elongated even after expression of the fusion protein, which indicated that sfGFP-AtFtsZ2-2MTS was unable to compensate for *ftsZ* depletion. In accord with this result, AtFtsZ2-2MTS failed to functionally complement the depleted endogenous EcFtsZ at both 37 °C and 22 °C on agar plates, and instead was toxic when overproduced at 37 °C (see Supplementary Fig. S4). However, sfGFP-AtFtsZ2-2MTS formed multiple Z ring-like structures and helical filaments in the elongated cells (Fig. 2B), and the full-length sfGFP-fused AtFtsZ2 was detected in immunoblotting assays (see Supplementary Fig. S3). The quantified results clearly demonstrated that the MTS noticeably and strongly contributed to the formation of Z ring-like structures composed of AtFtsZ2 (Fig. 2C). Intriguingly, in the EcFtsZ-depleted strain WM2767, sfGFP-AtFtsZ2 did not assemble into long filaments (Fig. 2B) as it did in the wild-type strain WM1074 (Fig. 1A), but instead formed short filaments and small dots. Because EcFtsZ was previously found to not heteropolymerize with AtFtsZ2^19^, the bundling of EcFtsZ and AtFtsZ2 homopolymers might be involved in this phenomenon. In *E. coli*, the nucleoid occlusion system prevents the Z ring from assembling on nucleoids, and thus the Z ring is formed between two segregated nucleoids^31^. To determine whether the Z ring-like structures of sfGFP-AtFtsZ2-2MTS localize between two segregated nucleoids in *E. coli*, we stained the nucleoids of *E. coli* WM2767 cells with DAPI (Fig. 2D): sfGFP-AtFtsZ2-2MTS formed multiple Z ring-like structures randomly at various positions over the nucleoids, suggesting that nucleoid occlusion did not function in the assembly of sfGFP-AtFtsZ2-2MTS in *E. coli* cells. However, these results indicate that if only anchored to the membrane, AtFtsZ2 *per se* can form Z ring-like structures in *E. coli* cells.

### Stromal protein ARC3 inhibits FtsZ assembly in *E. coli* cells as in chloroplasts

**Figure 3:**
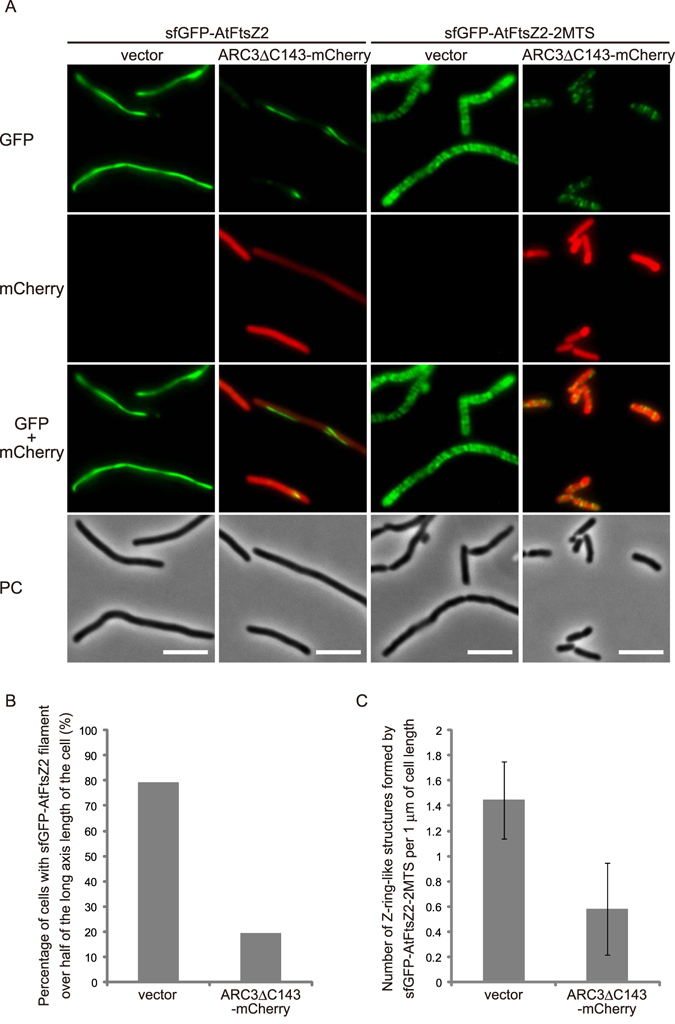
Stromal protein ARC3 exerts inhibitory effects on FtsZ assembly in *E. coli* cells. (**A**) Effect of ARC3 expression on AtFtsZ2 filaments and Z ring-like structures. Scale bar: 5 μm. (**B**) Quantification of ARC3 inhibition of AtFtsZ2 filament formation. The graph shows the percentage of cells containing sfGFP-AtFtsZ2 filaments that extended over half the cell length along the long axis. At least 118 elongated bacterial cells were investigated for the quantification. (**C**) Quantification of ARC3 inhibition of AtFtsZ2 Z ring-like structure formation. The graph shows the number of sfGFP-AtFtsZ2-2MTS Z ring-like structures per 1 μm of cell length. The mean and standard deviation were calculated from at least 89 cell samples.

ARC3, a chloroplast stromal protein that is absent in bacteria, interacts with both AtFtsZ1 and AtFtsZ2 and, through negative regulation of their assembly into filaments, spatially regulates chloroplast division. Hence, ARC3 is considered a functional homolog of bacterial MinC^18,24,32–34^. ARC3 interaction with AtFtsZs was shown to be inhibited by its C-terminal sequence of 143 amino acids including a membrane-occupation-and-recognition nexus (MORN) repeat motif, to which PARC6, the paralog of ARC6, binds directly and is suggested to promote ARC3 activity in the chloroplast division process^35^. Previous work conducted using the *S. pombe* expression system demonstrated that the fusion of a fluorescent protein to the C-terminus of ARC3∆C143 did not compromise the inhibitory function of ARC3^18^. To validate the ability of ARC3 to prevent AtFtsZ2 assembly into filaments in our *E. coli* expression system, sfGFP-AtFtsZ2 or sfGFP-AtFtsZ2-2MTS was coexpressed with mCherry-fused ARC3∆C143 (ARC3∆C143-mCherry), and following induction of ARC3∆C143-mCherry with NaSal, fluorescent signals in WM1074 cells were monitored (Fig. 3). Immunoblotting results confirmed that the sfGFP-AtFtsZ2 proteins and ARC3∆C143-mCherry were produced properly, although a large amount of cleaved mCherry was detected (see Supplementary Fig. S5). In both cases, we clearly observed that the formation of long filaments or Z ring-like structures was decreased in the presence of ARC3∆C143-mCherry (Fig. 3A). This inhibition of FtsZ assembly was also demonstrated by the results of quantitative analyses (Fig. 3B,C). Unlike in the case of AtFtsZ1 produced in yeast cells^18^, we could not confirm colocalization of AtFtsZ2 and ARC3 in *E. coli* cells. The function of ARC3 is proposed to be analogous to that of bacterial MinC, which transiently localizes to the cell pole and inhibits FtsZ polymerization. Given the high background fluorescence of cleaved mCherry (see Supplementary Fig. S5), it might be difficult to observe colocalization of ARC3 and AtFtsZ2 in *E. coli* cells. Nevertheless, these findings show that the negative regulator ARC3 functions in inhibiting AtFtsZ assembly in bacterial cells as in chloroplasts, which further supports the utility of this bacterial system for analyzing chloroplast division-related components.

### Chloroplast inner envelope membrane protein ARC6 accelerates FtsZ2 assembly into Z ring-like structures through interaction with the FtsZ2 C-terminus

**Figure 4:**
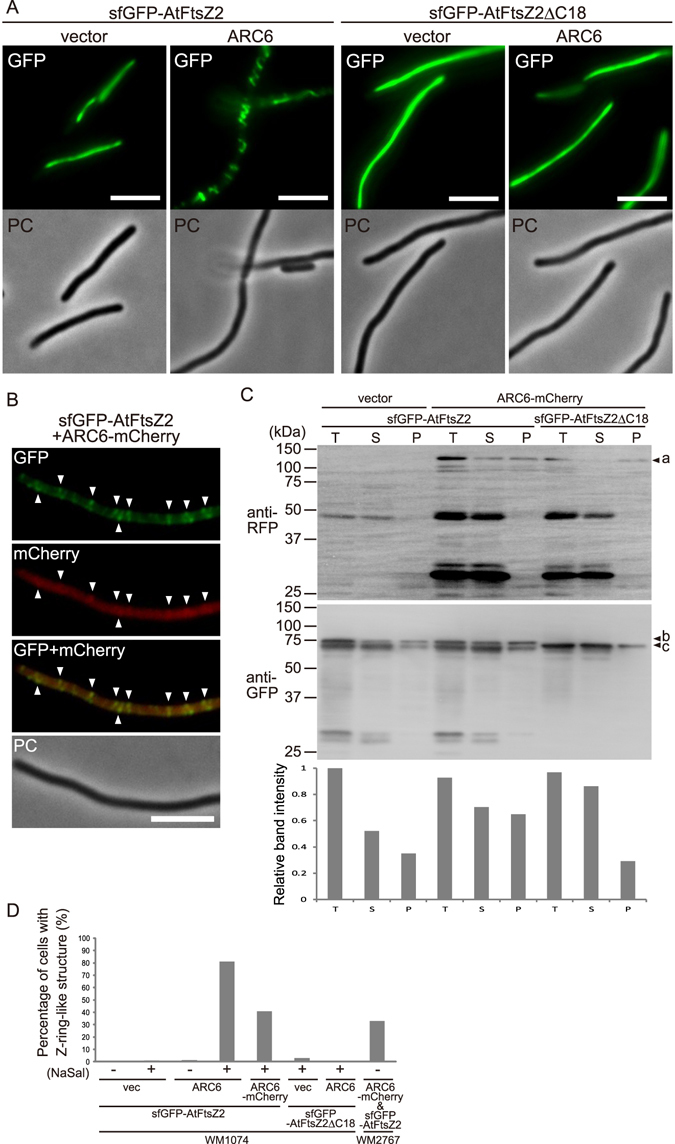
Chloroplast inner envelope membrane protein ARC6 accelerates FtsZ2 assembly into Z ring-like structures by interacting with the FtsZ2 C-terminus. (**A**) Effect of ARC6 expression on filament formation by AtFtsZ2 with or without the C-terminal ARC6-interacting sequence in the WM1074 strain. Scale bar: 5 µm. (**B**) Concurrent observation of Z ring-like structures formed by sfGFP-AtFtsZ2 and ARC6-mCherry in the WM1074 strain. Arrowheads indicate overlapping signals of AtFtsZ2 Z ring-like structure and ARC6. Scale bar: 5 µm. (**C**) Fractionation assay of the bacterial cell membrane. *E. coli* cells expressing sfGFP-AtFtsZ2 and ARC6-mCherry were fractionated by performing sonication and ultracentrifugation. Each protein was detected by immunoblotting with anti-GFP and anti-RFP antibodies. Arrowheads indicate the full-length protein bands of ARC6-mCherry (a), sfGFP-AtFtsZ2 (b) and sfGFP-AtFtsZΔC18 (c), the relative intensities of which were quantified using the software ImageJ (rsb.info.nih.gov/ij/). The band intensity of total lysate in the case of vector (pKG116) plus sfGFP-AtFtsZ2 was normalized to one. T, total lysate; S, supernatant (cytoplasmic) fraction; P, pellet (membrane) fraction. (**D**) Quantification of the Z ring-like structure signal of sfGFP-AtFtsZ2 coexpressed with ARC6 in the WM1074 and WM2767 strains. The graph shows the percentage of cells containing >1 Z ring-like structure. The numbers of elongated bacterial cells investigated for the quantification were 105 (WM1074/sfGFP-AtFtsZ2 & vector, −NaSal), 272 (WM1074/sfGFP-AtFtsZ2 & vector, +NaSal), 69 (WM1074/sfGFP-AtFtsZ2 & ARC6, −NaSal), 79 (WM1074/sfGFP-AtFtsZ2 & ARC6, +NaSal), 152 (WM1074/sfGFP-AtFtsZ2 & ARC6-mCherry, +NaSal), 35 (WM1074/sfGFP-AtFtsZ2∆C18 & vector, +NaSal), 60 (WM1074/sfGFP-AtFtsZ2∆C18 & ARC6, +NaSal), and 146 (WM2767/ARC6-mCherry & sfGFP-AtFtsZ2, −NaSal).

The membrane-tethering ability of AtFtsZ2, like that of bacterial FtsZ, largely contributes to the assembly of AtFtsZ2 into Z ring-like structures in bacterial cells. Osawa and Erickson successfully reconstituted a bacterial Z ring that was tethered to the membrane in liposomes by its natural anchoring partner FtsA^36^. In chloroplasts, the inner envelope membrane protein ARC6, which binds to the C-terminal conserved sequence of AtFtsZ2^37^, is a potential candidate to anchor Z ring to the membrane. AtFtsZ2 and ARC6 have been shown to interact directly by using a yeast two-hybrid system^15,37^. ARC6-GFP localized at chloroplast constriction sites, and *Arabidopsis arc6* mutants showed clear defects in Z ring formation and, consequently, chloroplast division^38^. These observations suggest that ARC6 is a potential anchoring partner of AtFtsZ2 to the membrane for Z ring formation, but direct evidence has not yet been reported. ARC6 is an ortholog of the division protein Ftn2/ZipN that is unique to cyanobacteria^38^, and *E. coli* expresses no ARC6 ortholog. In *E. coli* cells, sfGFP-AtFtsZ2 did not form Z ring-like structures (Figs 1A and 4A). Thus, we coexpressed sfGFP-AtFtsZ2 and ARC6 in the WM1074 strain and examined the AtFtsZ2 filament morphology (Fig. 4A, see Supplementary Fig. S6A). When ARC6 expression was induced using NaSal, the pattern of sfGFP-AtFtsZ2 fluorescence distribution changed drastically, from linear long filaments to Z ring-like structures and/or helices; by contrast, cells that were not induced with NaSal or carried an empty vector showed comparable sfGFP-AtFtsZ2 fluorescence patterns.

In a previous study, the results of a complementation assay with the Arabidopsis *arc6* mutant suggested that fusing a fluorescent protein to the ARC6 C-terminus did not disrupt ARC6 function in chloroplast division^38^. Therefore, we fused mCherry to the C-terminus of ARC6 (ARC6-mCherry) and the fusion protein was coexpressed with sfGFP-AtFtsZ2. Following induction of ARC6-mCherry with NaSal, concurrent fluorescence examination revealed that the ARC6-mCherry signal colocalized with sfGFP-AtFtsZ2 Z ring-like structures (Fig. 4B), although the mCherry signal was also detected in the cytoplasm probably because of cleavage of ARC6-mCherry (see below). To test whether the morphological change of sfGFP-AtFtsZ2 from linear filaments to Z ring-like structures depends on the direct interaction between AtFtsZ2 and ARC6, we created and expressed an AtFtsZ2 truncation mutant lacking the C-terminal ARC6-interacting sequence (sfGFP-AtFtsZ2∆C18)(see Supplementary Fig. S1), and our results showed that the linear-filament distribution of sfGFP-AtFtsZ2∆C18 was unaffected by NaSal-induced ARC6 expression (Fig. 4A). ARC6 protrudes into the chloroplast inner envelope membrane with its C-terminus and directly interacts with AtFtsZ2 through its N-terminal region in the stromal side^37,38^. Thus, we performed membrane-fractionation assays to test whether ARC6 binds to the *E. coli* cell membrane and subsequently anchors sfGFP-AtFtsZ2 to the membrane. After cell fractionation through sonication and ultracentrifugation, full-length ARC6-mCherry was detected in the membrane fraction but the truncated form and cleaved mCherry were detected only in the soluble fraction, which confirmed ARC6 targeting to the *E. coli* membrane (Fig. 4C). Concomitantly, a large amount of sfGFP-AtFtsZ2 was also detected in the membrane fraction, whereas the amount of sfGFP-AtFtsZ2∆C18 in the membrane fraction was clearly decreased to the control level. This result was further corroborated by quantification of the band intensity of each protein. In the case of sfGFP-AtFtsZ2, the total amount of full-length and truncated product bands was quantitated. The results confirmed the membrane anchoring of sfGFP-AtFtsZ2 through direct interaction with ARC6 (Fig. 4C).

To assess the effect of endogenous EcFtsZ on the formation of ARC6-tethered AtFtsZ2 Z ring-like structures, we constructed a plasmid carrying both *ARC6-mCherry* and *sfGFP-AtFtsZ2* in tandem under the control of a single IPTG-inducible promoter and introduced it into the EcFtsZ-depleted WM2767 strain. Our results showed that even when endogenous EcFtsZ was heavily depleted, sfGFP-AtFtsZ2 frequently formed Z ring-like structures in the presence of ARC6, which indicates that ARC6-tethered AtFtsZ2 *per se* can form Z ring-like structures in bacterial cells (see Supplementary Fig. S6B). However, we could not detect ring-like structures corresponding to ARC6, possibly because of the strong background fluorescence of mCherry. Moreover, immunoblotting analysis revealed that ARC6-mCherry was largely cleaved, yielding mCherry and non-fused ARC6 (see Supplementary Fig. S6C). Thus, we concluded that both the non-fused ARC6 and full-length ARC6-mCherry recruited AtFtsZ2 to the cytoplasmic membrane. The effect of ARC6 on Z ring-like structure formation was further supported by the results of quantitative analyses, while the fusing mCherry works negatively on this effect (Fig. 4D). The expression of full-length EcFtsZ, sfGFP-AtFtsZ2, and ARC6-mCherry was confirmed through immunoblotting (see Supplementary Fig. S6C). Collectively, these results strongly suggest that AtFtsZ2 is tethered to the membrane through direct interaction with ARC6 and assembles into Z ring-like structures. Bolstering these findings, ARC6 exerted a markedly smaller effect on the fluorescence pattern of AtFtsZ2-sfGFP (C-terminal fusion) as compared with that of sfGFP-AtFtsZ2 (N-terminal fusion), probably due to the interaction between AtFtsZ2 and ARC6 being inhibited, to some degree, by sfGFP fusion at the C-terminus of AtFtsZ2 (see Supplementary Fig. S7).

### FtsZ1 affects the filament morphology of FtsZ2 in *E. coli* cells

**Figure 5:**
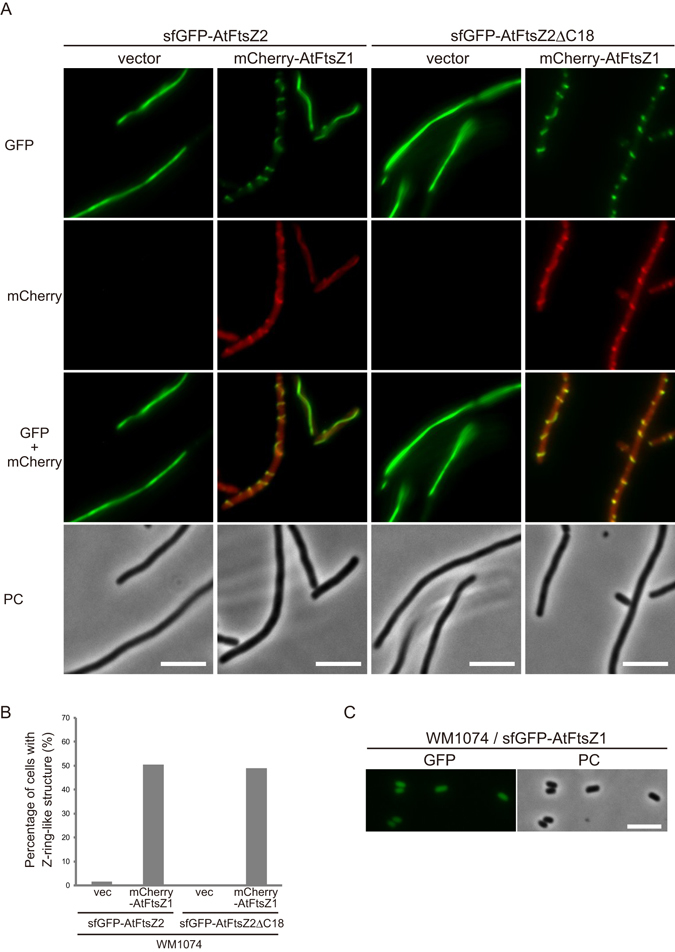
FtsZ1 affects the filament morphology of FtsZ2 in *E. coli* cells. (**A**) Effect of AtFtsZ1 expression on the filaments of AtFtsZ2 with or without the partially conserved C-terminal sequence in the WM1074 strain. Scale bar: 5 µm. (**B**) Quantification of the Z ring-like signal of sfGFP-AtFtsZ2 expressed with or without mCherry-AtFtsZ1. The graph shows the percentage of cells containing >1 Z-ring-like structure. The numbers of elongated bacterial cells investigated for the quantification were 60 (WM1074/sfGFP-AtFtsZ2 & vector, +NaSal), 107 (WM1074/sfGFP-AtFtsZ2 & mCherry-AtFtsZ1, +NaSal), 49 (WM1074/sfGFP-AtFtsZ2∆C18 & vector, +NaSal) and 90 (WM1074/sfGFP-AtFtsZ2∆C18 & mCherry-AtFtsZ1, +NaSal). (**C**) Fluorescence image of sfGFP-AtFtsZ1 expressed by itself in the WM1074 strain. Scale bar: 5 µm.

Although the chloroplast Z ring in *A. thaliana* consists of AtFtsZ1 and AtFtsZ2, the filament morphology of AtFtsZ2 is more dominant than that of AtFtsZ1^18,19^. AtFtsZ1 harbors no ARC6-interacting sequence at its C-terminus and is proposed to act as a regulator of Z ring turnover and remodeling^18,22,23^ (see Supplementary Fig. S1). The formation of the ring-shaped filament and Z ring-like structures of MTS-tagged AtFtsZ2 in *P. pastoris* and *E. coli*, respectively, strongly supports the function of AtFtsZ2 as the backbone of the AtFtsZ filament^19^ (Fig. 2). This view is also supported by the observation that chloroplasts in the *Arabidopsis ftsZ1* mutant still exhibited a single mid-plastid constriction, suggesting that AtFtsZ2 is functional in the *ftsZ1* mutant^24^. Nevertheless, like the *ftsZ2* mutant, the *ftsZ1* mutant displays chloroplast division defects such as a reduced number of enlarged chloroplasts, which indicates the crucial role of AtFtsZ1 in chloroplast division^15^. To investigate the effect of AtFtsZ1 on the AtFtsZ2 filaments formed in *E. coli* cells, we coexpressed sfGFP-AtFtsZ2 and AtFtsZ1 N-terminally fused to mCherry (mCherry-AtFtsZ1) and then monitored their fluorescence distribution. Following induction of mCherry-AtFtsZ1 with NaSal, unexpectedly, the long filaments of sfGFP-AtFtsZ2 in WM1074 cells were morphologically changed into helical and/or Z ring-like structures in the presence of mCherry-AtFtsZ1 (Fig. 5A). The mCherry-AtFtsZ1 signal clearly overlapped with the sfGFP-AtFtsZ2 signal, which implies heteropolymerization of AtFtsZ1 and AtFtsZ2. AtFtsZ1 possesses no C-terminal sequence that can bind to any membrane-anchoring factors, either FtsA/ZipA in bacteria or ARC6 in chloroplasts^18,22,23^ (see Supplementary Fig. S1), whereas AtFtsZ2, which contains the C-terminal ARC6-interacting sequence, did not form any Z ring-like structures in wild-type WM1074 cells in the absence of ARC6 (Figs 1A, 2C, and 4A,E). One possibility is that the C-terminal domain of AtFtsZ2 in AtFtsZ1/AtFtsZ2 heteropolymers acquired the ability to interact with FtsA/ZipA in *E. coli* because the C-terminal domain of FtsZ serves as dock for proteins that regulate FtsZ assembly. These proteins include FtsA, ZipA, MinC, and SlmA. To test the effects of AtFtsZ2 C-terminus on this phenomenon, we coexpressed sfGFP-AtFtsZ2∆C18 and mCherry-AtFtsZ1, but again observed that the Z ring-like signals of sfGFP-AtFtsZ2∆C18 overlapped with the mCherry-AtFtsZ1 signals, which suggests that the AtFtsZ1-induced morphological change of AtFtsZ2 filaments does not depend on the C-terminus of AtFtsZ2 (Fig. 5A,B). We clearly detected full-length EcFtsZ, sfGFP-AtFtsZ2, and mCherry-AtFtsZ1 in immunoblotting assays (see Supplementary Fig. S8). These results suggest that the AtFtsZ1/AtFtsZ2 heteropolymer can form Z ring-like structures independently of the EcFtsZ-FtsA/ZipA interaction in *E. coli* cells. AtFtsZ2 has been reported not to form heteropolymers with EcFtsZ^19^, but whether AtFtsZ1 and EcFtsZ can assemble into heteropolymers remains unclear. However, when sfGFP-AtFtsZ1 was expressed independently, it dispersed uniformly in WM1074 cells with regular cell lengths, which implies that AtFtsZ1 does not significantly interact with EcFtsZ (Fig. 5C). How AtFtsZ1/AtFtsZ2 heteropolymers form Z ring-like structures without ARC6 in *E. coli* cells remains to be elucidated. However, our results clearly indicate that AtFtsZ1 positively affects the morphological properties of AtFtsZ2 filaments and thereby promotes the formation of Z ring-like structures.

## Discussion

We have presented here the first example of chloroplast Z ring reconstitution in a heterologous expression system developed using bacteria, the evolutionary ancestors of the chloroplast. Given that microbial systems offer the practical advantages of both axenic culture and genetic accessibility, establishing bacterial heterologous expression systems for reconstituting the cellular events of plant cells is crucial. While we were developing our *E. coli* system, Yoshida *et al*. reported a similar reconstitution system with *P. pastoris*^19^. Thus, the yeasts *S. pombe* and *P. pastoris* and a bacterium, *E. coli*, have now been used for heterologous expression of chloroplast FtsZs and other chloroplast division-related proteins owing to the complexities faced in *in planta* analysis^18,19,25^ (and this study), while there are some inverse examples, where the bacterial FtsZ has been heterologously produced in eukaryotic cells^39,40^. Yeast reconstitution systems have shown the value of using heterologous expression systems for studying the inherent properties of chloroplast division-related proteins, particularly filament and ring formation by FtsZs in eukaryotic cellular environments. However, our novel *E. coli* system provides a new approach for dissecting and uncovering the chloroplast division process in the stromal side, which is topologically equivalent to the bacterial cytosol, although the possible post-translational modifications of AtFtsZs reported by Karamoko *et al*.^41^ would not occur in bacteria.

In *E. coli* wild-type strain cells, sfGFP-AtFtsZ2, but not AtFtsZ2-sfGFP, assembled into long filaments (Fig. 1A, see Supplementary Fig. S2A). Thus far, almost all studies on bacterial and chloroplast FtsZs have used C-terminal fluorescent-protein fusions to monitor the subcellular localization of each FtsZ. Bacterial FtsZ fused with GFP at its C-terminus functions properly at least in assembly into Z rings, although it cannot fully complement *ftsZ* mutants^21^. In chloroplasts, the *Arabidopsis ftsZ1* knockout mutant can be complemented by C-terminal fluorescent-protein fusions of AtFtsZ1 but not of *ftsZ2* mutant by C-terminal fusion proteins of AtFtsZ2, which further supports the notion that AtFtsZ2 and AtFtsZ1 function as the filament backbone and regulator, respectively^18^. These complementation defects appear to result partially from the fluorescent-protein tag blocking the membrane tethering of the proteins by their C-terminal conserved peptides^18,42^. The difference in filamentation ability between the N- and C-terminal sfGFP fusions of AtFtsZ2 might consistently reflect the importance of the C-terminal sequence of FtsZ proteins for their function. In *A. thaliana*, AtFtsZ2 tagged C-terminally with a fluorescent protein is assembly competent, which suggests that the other division-related proteins in chloroplasts exert supportive effects^18,34^.

Temperature and bacterial growth phase can also critically affect AtFtsZ2 in our bacterial expression system. AtFtsZ2-sfGFP assembled into large spots, presumably inclusion bodies, at 37 °C but not at 22 °C (see Supplementary Fig. S2A), whereas cells carrying the gene *sfGFP-AtFtsZ2* frequently appeared atypically white and exhibited little or no fluorescence, and large sfGFP-AtFtsZ2 aggregates were not detected at 37 °C (Fig. 1A). Conversely, both EcFtsZ-GFP and sfGFP-EcFtsZ assembled into typical Z rings at both 37 °C and 22 °C (Fig. 1A, see Supplementary Fig. S2A). *A. thaliana* is a winter annual plant that grows optimally at 22 °C–23 °C. Thus, AtFtsZ2 might be optimized to function more effectively at around 22 °C than at 37 °C. On the other hand, in stationary-phase bacterial cells incubated at 37 °C, we detected higher expression levels of sfGFP-AtFtsZ2 than that of sfGFP-EcFtsZ, and this might explain the toxic effect on *E. coli* cells. However, further investigation is required to confirm the protein’s temperature dependency. We observed that sfGFP-AtFtsZ2 efficiently assembled into filaments only in the stationary phase of *E. coli* wild-type strain (Fig. 1A). Reduced dynamics and a lower turnover rate of AtFtsZ2 as compared with those of EcFtsZ is one possible explanation for this phenomenon^18^. Another possibility is that there is less competition with actively assembling EcFtsZ during the stationary phase.

Regardless of the temperature, we occasionally detected faint bands at around 55 kDa, which are potentially associated with the dimerization of sfGFP molecules (Fig. 1C, see Supplementary Fig. S3). However, the overall behavior of sfGFP-AtFtsZ2 with/without MTS or other chloroplast components in this study, particularly the formation of Z ring-like structures, strongly suggest that we observed the inherent characteristics of this protein rather than artifacts by the possible dimerization of sfGFP.

Only one study has reported functional complementation of *E. coli* cell division by chloroplast FtsZ^43^: FtsZ from the pea plant (*Pisum sativum*) was shown to correct the thermosensitive defect of *E. coli ftsZ* mutant. However, we found that AtFtsZ2 did not rescue the colonization defects of the *E. coli* FtsZ-depletion mutant at either 37 °C or 22 °C, even though the extra N-terminal region of AtFtsZ2 was deleted and its C-terminal region was tagged with 2MTS (see Supplementary Fig. S4). At 22 °C, sfGFP-AtFtsZ2-2MTS formed Z ring-like structures in *E. coli* cells, and *E. coli* cell division occurred normally with sfGFP-EcFtsZ localized correctly at mid-cell (Figs 1A and 2A,B); therefore, we concluded that AtFtsZ2 could not functionally complement EcFtsZ in *E. coli* cells. To date, no intact form of a foreign bacterial FtsZ has been observed to complement EcFtsZ function^44^. Thus, this issue remains controversial.

Membrane tethering is required for Z ring-like structure formation^4,19,36^ (Figs 2 and 4). The FtsA/ZipA-interacting sequence of EcFtsZ is partially conserved at the AtFtsZ2 C-terminal region (see Supplementary Fig. S1), but MTS-lacking sfGFP-AtFtsZ2 forms only long filaments in *E. coli* cells (Fig. 1A); this finding indicates that AtFtsZ2 cannot interact with *E. coli* FtsA/ZipA and thus cannot be targeted to the bacterial membrane. The addition of 2MTS to the AtFtsZ2 C-terminus drastically alters the filament morphology, from linear to ring-shaped, regardless of the presence or absence of EcFtsZ (Fig. 2A,B). These rings are perpendicular to the membrane plane, to some degree, and resemble the Z rings immunostained in elongated bacterial cells of both thermosensitive *ftsZ* and *ftsZ*-depletion mutants^42^. Given that the bacterial cell-division machinery correctly localizes at the inter-chromosomal space, the Z ring-like structures of sfGFP-AtFtsZ2-2MTS detected over the bacterial chromosome might not be incorporated into the cell-division machinery of *E. coli*. Collectively, our results indicate that membrane-targeted AtFtsZ2 can assemble into Z ring-like structures as a filament backbone in the absence of EcFtsZ and other chloroplast division-related proteins in our bacterial expression system. The partly slanted AtFtsZ2 rings might be due to the lack of connection to the peptidoglycan machinery^45^. Here, we did not observe the constriction of *E. coli* cells by the expressed sfGFP-AtFtsZ2-2MTS (Fig. 2B). Bacteria feature a peptidoglycan-containing cell wall that provides rigidity to the cell and maintains the shape of each bacterium. Because the constricting ring of MTS-tagged AtFtsZ2 expressed in spherical *P. pastoris* cells has been proposed to slide along the membrane from the middle region to one end of the cell^19^, we suggest that the cell-wall integrity of *E. coli* restricts the constriction of Z ring-like structures formed by sfGFP-AtFtsZ2-2MTS in our bacterial system. Notably, whereas long filaments of sfGFP-AtFtsZ2 were observed only when endogenous EcFtsZ was present (Figs 1A and 2B), multiple Z ring-like structures of sfGFP-AtFtsZ2-2MTS were formed even in the EcFtsZ-depleted strain (Fig. 2B), which suggests that EcFtsZ exerts a supportive effect particularly on AtFtsZ2 filamentation ability. The long filaments detected in wild-type cells are possibly generated by bundling of EcFtsZ and AtFtsZ2 homopolymers because EcFtsZ cannot heteropolymerize with AtFtsZ2^19^.

To reconstitute the inner membrane assembly complex of the chloroplast division machinery in bacteria, it is critical to introduce other regulatory components into bacterial cells together with AtFtsZ2 and accurately evaluate their functions. In *S. pombe*, ARC3 colocalizes with AtFtsZ1 and inhibits its polymerization, which indicates that ARC3 expressed in yeast cells acts as a negative regulator of AtFtsZ1, as in chloroplasts^18^. ARC3 also interacts with AtFtsZ2 in a yeast two-hybrid system, and AtFtsZ2 filament assembly is inhibited in *A. thaliana* ARC3-overexpressing plants^24^. Consistently, nonbacterial ARC3 expression potently lowered the filament length of sfGFP-AtFtsZ2 and reduced the number of Z ring-like structures formed by sfGFP-AtFtsZ2-2MTS in bacterial cells (Fig. 3). Here, the AtFtsZ2 Z ring-like structures were forcibly tethered to the membrane by the MTS, and ARC3 strongly inhibited AtFtsZ2 filamentation, and thus the number of Z ring-like structures formed might have been reduced due to the inhibition of AtFtsZ2 filament assembly by ARC3. Notably, the cells expressing both AtFtsZ2-2MTS and ARC3 were much shorter than those expressing only AtFstZ2-2MTS were. This is consistent with the reduced filamentation of AtFtsZ2-2MTS by ARC3. On the other hand, ARC3-expressing cells with short filaments of non-membrane-targeted AtFtsZ2 were still elongated. Although the underlying mechanistic details remain unclear, membrane-tethered short filaments of AtFtsZ2-2MTS appear to be less toxic for *E. coli* cell division than free-floating filaments of AtFtsZ2.

Direct targeting of FtsZ proteins to the membrane has not yet been reported in either bacteria or chloroplasts, but FtsZ is known to be recruited to the membrane by other transmembrane or membrane-associated proteins such as FtsA/ZipA in *E. coli*^20,21,29,30,36,46,47^. Artificial membrane targeting of EcFtsZ and AtFtsZ2 through C-terminal tagging with MTS confers the proteins with the ability to form membrane-tethered Z ring-like structure^4,19^ (Fig. 2). However, no evidence reported thus far indicates that the AtFtsZ2-interacting protein ARC6 directly tethers chloroplast FtsZ to the membrane component for Z ring formation. In our bacterial system, ARC6 colocalized with AtFtsZ2 and drastically altered its filament morphology, from linear filaments to Z ring-like structures (Fig. 4, see Supplementary Fig. S6B). This Z ring-like structure formation and the membrane attachment of AtFtsZ2 strongly depended on the C-terminal ARC6-interacting sequence of AtFtsZ2, which indicates that ARC6-tethered AtFtsZ2 behaves like AtFtsZ2-2MTS, an artificial membrane-targeting protein, and assembles into multiple Z ring-like structures. Conversely, the Z ring-like structures formed by AtFtsZ2 and ARC6 in bacterial cells reflect the natural condition of the chloroplast Z ring, which concurrently demonstrates that ARC6 functions as a genuine membrane-anchoring partner of AtFtsZ2.

Recently, ARC6 was reported to be capable of stabilizing AtFtsZ2 polymers in yeast cells independently of its Z ring-tethering function^25^; the stromal region of ARC6 was observed to lower the turnover rate of AtFtsZ2 polymers. This phenomenon might affect, at least partially, the formation of multiple Z ring-like structures of AtFtsZ2 in ARC6-expressing bacterial cells, although the formation of Z ring-like structures is correctly due to membrane tethering through the AtFtsZ2-ARC6 interaction. The crystal structure of the conserved domain in the intermembrane space region of ARC6 has been solved, but the requirement of this region for the interaction with the outer envelope membrane protein PDV2 and the coordination between the inner and outer envelope division machineries remain unclear^48^. Solving the crystal structure of the stromal region of ARC6, particularly of co-crystals with AtFtsZ2, will provide additional insights into the ARC6-AtFtsZ2 interaction and the membrane tethering of chloroplast Z rings.

In plants and algae, FtsZ1 and FtsZ2 function together in forming the Z ring in the chloroplast division machinery^3,14,49^. Despite the high identity and similarity in their amino acid sequence, these two FtsZ proteins of *A. thaliana* play distinct roles in chloroplast division^14,15,18^. When AtFtsZ1 and MTS-tagged AtFtsZ2 are coexpressed, AtFtsZ1 forms ring structures together with AtFtsZ2 in *P. pastoris*^19^. Intriguingly, in *E. coli* cells, AtFtsZ1 supported the formation of Z ring-like structures of AtFtsZ2 lacking the MTS tag (Fig. 5), but when expressed separately, AtFtsZ1 itself did not assemble into filaments or ring-like structures in our bacterial system. These findings and the previous observation that AtFtsZ1 assembled into ring-like structures without MTS-tagging in *P. pastoris*^19^ together suggest that further characterization of AtFtsZ1 is required, with a particular focus on its direct interaction with membrane components. Notably, unlike sfGFP-AtFtsZ2, sfGFP-AtFtsZ1 expressed in *E. coli* did not produce division-defective effects, which supports the notion of AtFtsZ2 dominance over AtFtsZ1 as a core component of the Z ring filament.

To date, heterologous expression systems using *P. pastoris*^19^ and *E. coli* (this study) have successfully formed clear rings/bands of AtFtsZ2, which is tethered to the membrane by MTS. Additionally, in our system, membrane tethering by MTS could be replaced by co-production of ARC6. We strongly believe that AtFtsZ2, when anchored to the membrane, exhibits an intrinsic property to form such rings, similar to EcFtsZ. Purified EcFtsZ-MTS can form distributed ring-like structures in tubular liposomes^4^. We speculate that AtFtsZ2-2MTS also forms similar structures *in vitro*. Thus far, we have not been able to purify AtFtsZ2-2MTS; therefore, we do not know if AtFtsZ2-2MTS can form Z ring-like structures within liposomes in the same way as EcFtsZ-MTS^4^. If AtFtsZ2-2MTS also forms ring-like structures similar to EcFtsZ-MTS, then FtsZ, in general, may have the ability to attach to a particular membrane curvature.

Plant chloroplasts are surrounded by two envelope membranes, and the inner envelope membrane protein ARC6 plays the crucial role of interacting with the outer envelope membrane protein PDV2 and thereby enabling coordination between the inner (stromal) and outer (cytosolic) division machinery^50^. The successful reconstitution of the membrane-tethered chloroplast Z ring through its natural partner ARC6 in *E. coli* cells suggests that the bacterial cell—the chloroplast ancestor—can serve as a novel experimental system for analyzing the stromal components in the chloroplast divisome complex. The evolutionary distance is high between *E. coli* and cyanobacteria, which are the direct bacterial ancestors of the chloroplast, and certain stromal proteins of the chloroplast divisome, including ARC6 and ARC3, are absent in *E. coli*; this enhances the value of this bacterium for use in the heterologous system. In addition to our observation of AtFtsZ2 Z ring-like structure formation, our results showing that ARC3 and ARC6, the negative and positive regulators in Z ring formation, respectively, function properly in *E. coli* cells strongly indicate the usefulness of bacterial cells for analyzing the chloroplast division machinery.

## Methods

### Bacterial strains and growth media

All strains used were derivatives of *E. coli* K-12. Strains WM1074^51^ and WM2767^52^ were kindly provided by Dr. Margolin (University of Texas Houston). WM1074 is a wild-type strain, and WM2767 lacks chromosomal *ftsZ* but harbors a salicylate-inducible *ftsZ*-expressing plasmid, pWM2765. Cells were grown in L broth (1% bactotryptone, 0.5% yeast extract, 0.5% NaCl) at the mentioned temperatures. When necessary, kanamycin (20 μg/mL), ampicillin (100 μg/mL), chloramphenicol (20 μg/mL), and NaSal (10 μM) were added to the cultures. These compounds were purchased from Wako (Osaka, Japan). In liquid culture, each protein was expressed from the pDSW204^53^-based plasmid without any inducers, except in the case of cells with plasmid harboring *sfGFP-AtFtsZ2* and *ARC6-mCherry* in tandem. For the induction of each protein from pKG116^54^-based plasmid, NaSal was added at the beginning of the second culture. Sampling of the cells was performed during either the exponential (OD_600_ < 1.0) or stationary phase (OD_600_ > 1.8). In the complementation assay, IPTG (100 μM) was added to the plate medium.

### Plasmids

All plasmids used in this study are listed in Table S1. All DNA sequences of chloroplast division proteins used in this study lacked the sequences encoding cTPs predicted by ChloroP (http://www.cbs.dtu.dk/services/ChloroP/) except AtFtsZ1. The cTP of AtFtsZ1 was removed following a previous report^18^. All plasmids carrying *FtsZ2* of *A. thaliana* and *ftsZ* of *E. coli* were cloned into pDSW204, which carries the ampicillin resistance gene (*bla*). To generate pRU194 and pRU200, PstI-HindIII fragments of PCR-amplified *sfGFP* and *sfGFP*-*2MTS* were introduced into the corresponding sites in pDSW204, respectively. SacI-BamHI fragments of *FtsZ2-1* and *FtsZ2-1*∆*N* lacking the stop codon were amplified from *A. thaliana* cDNA and introduced into the corresponding sites of pRU194, which generated pRU310 and pRU711, respectively. To obtain pRU296, the SacI-BamHI fragment of *FtsZ2-1* lacking the stop codon was introduced into the corresponding site of pRU200. For the complementation assays, the SacI-BamHI fragments of *FtsZ2-1* and *FtsZ2-1*∆*N* including the stop codon were amplified and introduced into the corresponding sites of pRU200, which yielded pRU329 and pRU712, respectively. The SacI-HindIII fragments of *ftsZ*, *FtsZ2-1*-*2MTS*, and *FtsZ2-1*∆*N*-*2MTS* were amplified and introduced into the corresponding sites of pDSW204, which resulted in pRU731, pRU613, and pRU722, respectively. To generate pRU874 and pRU876, the EcoRI-SacI fragment of *sfGFP* lacking the stop codon was amplified and introduced into the corresponding sites of pRU329 and pRU613, respectively. The SacI-HindIII fragments of PCR-amplified *FtsZ2-1*∆*C18* and *ftsZ* of pRU731 were replaced with the corresponding regions of pRU874, which yielded pRU888 and pRU878, respectively. To generate pRU972, the SacI-BamHI fragment of *FtsZ1* including the stop codon was amplified and introduced into the corresponding site of pRU200. Next, the EcoRI-SacI fragment of *sfGFP* was introduced into corresponding site. All plasmids carrying only *ARC6*, *ARC3*, or *FtsZ1* of *A. thaliana* were derivatives of pKG116, which harbors the chloramphenicol resistance gene (*cat*). The NdeI-KpnI fragment of *ARC6* was amplified from *A. thaliana* cDNA and introduced into the corresponding sites of pKG116, which yielded pRU325. To generate pRU394, the KpnI-XhoI fragment of *mCherry* was amplified and introduced into the corresponding site of pKG116. Next, the NdeI-KpnI fragments of *ARC6* and *ARC3*∆*C143* lacking the stop codon were amplified from *A. thaliana* cDNA and introduced into the corresponding sites of pRU394, which resulted in pRU889 and pRU886, respectively. To generate pRU930, the NdeI-KpnI fragment of *FtsZ1* amplified from *A. thaliana* cDNA ﻿was introduced into the corresponding sites﻿ of pKG116 and then﻿﻿﻿ the NdeI-NdeI fragment of *mCherry* lacking the stop codon was amplified and introduced into the corresponding sites of the resulting plasmid. For concurrent expression of sfGFP-AtFtsZ2 with ARC6-mCherry under the control of a single promoter, the NcoI-NcoI fragment of *ARC6-mCherry* was amplified using pRU889 as a template and the fragment was then introduced into the corresponding site of pRU874 to obtain pRU957.

### Immunoblotting

Sample proteins were analyzed by polyacrylamide gel electrophoresis using 12% polyacrylamide gel and transferred onto polyvinylidene difluoride membranes (EMD Millipore). Immunoblotting was performed using anti-GFP (1:3,000) and anti-RFP (1:3,000) antibodies (MBL, Nagoya, Japan) as the primary antibodies. Anti-Rabbit IgG AP-linked antibody (1:3,000, CST) was used as the secondary antibody. The signals were visualized with DDAO phosphate (Molecular Probes, Massachusetts, USA) and detected using Typhoon FLA 9500 (GE Healthcare Life Sciences, Chicago, Illinois USA).

### Microscopy

Cells were mounted on M9-agarose pads and examined using an Axio Imager A2 (Zeiss, Germany) equipped with a cooled CCD camera (Axiocam MR; Zeiss, Oberkochen, Germany). Images were acquired and processed using ZEN (Zeiss), Photoshop (Adobe), and ImageJ.

### Cell fractionation

*E. coli* cells expressing sfGFP-AtFtsZ2 and ARC6-mCherry were harvested and suspended in Sonication buffer (50 mM Tris-HCl, pH 8.8, containing one tablet of protease-inhibitor cocktail [Roche, Basel] per 10 mL). After freezing at −30 °C, cells were lysed by sonicating them and then the supernatants were collected, following which ultracentrifugation at 32,000 rpm for 30 min at 4 °C (S55A2 angle rotor, Hitachi KOKI, Tokyo, Japan) was performed to divide the samples into membrane and cytoplasmic fractions.

## Electronic supplementary material

Supplementary Information
