# Author Correction: ARC6-mediated Z ring-like structure formation of prokaryote-descended chloroplast FtsZ in *Escherichia coli*

**DOI:** 10.1038/s41598-018-23160-5

**Published:** 2018-03-15

**Authors:** Hiroki Irieda, Daisuke Shiomi

**Affiliations:** 1 Department of Life Science, College of Science, Rikkyo University, Tokyo, 171-8501 Japan; 2 Present Address: Academic Assembly, Institute of Agriculture, Shinshu University, 8304 Minamiminowa, Kami-Ina, Nagano, 399-4598 Japan

Correction to: *Scientific Reports* 10.1038/s41598-017-03698-6, published online 14 June 2017

This Article contains errors in Reference 19 which was incorrectly given as:

“Yoshida, Y., Mogi, Y., TerBush, A. D. & Osteryoung, K. W. Chloroplast FtsZ assembles into a contractible ring via tubulin-like heteropolymerization. *Nat. Plants* **2**, 16095 (2016).”

The correct reference is listed below:

“Yoshida, Y., Kuroiwa, H., Misumi, O., Nishida, K., Yagisawa, F., Fujiwara, T., Nanamiya, H., Kawamura, F. & Kuroiwa, T. Isolated chloroplast division machinery can actively constrict after stretching. *Science* **313**,1435–1438 (2006)”

