# Supplementary material for: ARC6-mediated Z ring-like structure formation of prokaryote-descended chloroplast FtsZ in Escherichia coli
Source: Sci Rep. 2017 Jun 14;7:3492. doi: 10.1038/s41598-017-03698-6 (PMC5471200; doi:10.1038/s41598-017-03698-6)
Supplement: Supplementary file 1 — Supplementary Information [file 41598_2017_3698_MOESM1_ESM.pdf]

Supplementary Information

ARC6-mediated Z ring-like structure formation of prokaryote-descended chloroplast FtsZ in *Escherichia coli*

Hiroki Irieda† and Daisuke Shiomi*

Department of Life Science, College of Science, Rikkyo University, Tokyo 171-8501, Japan.

†Present address: Academic Assembly, Institute of Agriculture, Shinshu University, 8304 Minamiminowa, Kami-Ina, Nagano 399-4598, Japan.

*Corresponding author: Daisuke Shiomi

Tel: +81-3-3985-2401

Fax: +81-3-3985-2401

E-mail: dshiomi@rikkyo.ac.jp

Running title: Membrane tethering of chloroplast Z ring via ARC6

Table S1. Plasmids used in this study.

Plasmids	Description	Source/reference
pDSW204		Weiss *et al.* , 1999
pRU194	*sfGFP*	This study
pRU200	*sfGFP-2MTS*	This study
pRU329	*AtFtsZ2-1*	This study
pRU613	*AtFtsZ2-1-2MTS*	This study
pRU310	*AtFtsZ2-1-sfGFP*	This study
pRU296	*AtFtsZ2-1-sfGFP-2MTS*	This study
pRU712	*AtFtsZ2-1* Δ*N*	This study
pRU722	*AtFtsZ2-1* Δ*N-2MTS*	This study
pRU711	*AtFtsZ2-1* Δ*N-sfGFP*	This study
pRU731	*ftsZ*	This study
pRU874	*sfGFP-AtFtsZ2-1*	This study
pRU888	*sfGFP-AtFtsZ2-1* Δ*C18*	This study
pRU876	*sfGFP-AtFtsZ2-1-2MTS*	This study
pRU878	*sfGFP-ftsZ*	This study
pRU972	*sfGFP-AtFtsZ1*	This study
pRU957	*ARC6-mCherry* &*sfGFP-AtFtsZ2-1*	This study
pKG116		Buron-Barral *et al.* , 2006
pRU394	*mCherry*	This study
pRU325	*ARC6*	This study
pRU889	*ARC6-mCherry*	This study
pRU886	*ARC3* Δ*C143-mCherry*	This study
pRU930	*mCherry-AtFtsZ1*	This study

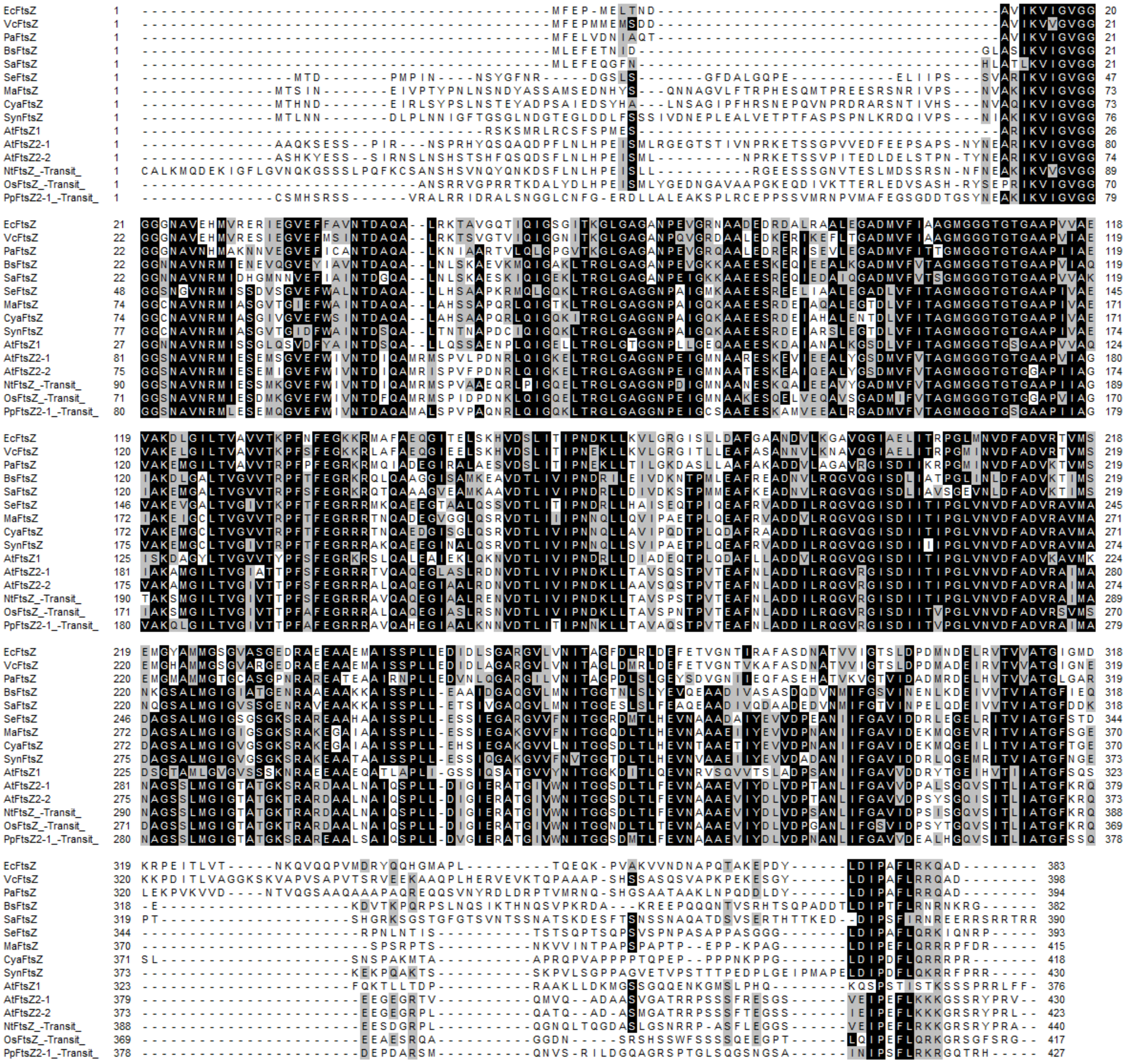

Figure S1. Amino acid sequence alignment of FtsZ homologues in bacteria and plants. FtsZ homologues of *Escherichia coli* (Ec), *Vibrio cholerae* (Vc), *Pseudomonas aeruginosa* PAO1 (Pa), *Bacillus subtilis* (Bs), *Staphylococcus aureus* (Sa), *Synechococcus elongatus* PCC 7942 (Se), *Microcystis aeruginosa* PCC 7806 (Ma), *Cyanothece* sp. PCC 7822 (Cya), *Synechocystis* sp. PCC 6803 (Syn), *Arabidopsis thaliana* (At), *Nicotiana tabacum* (Nt), *Oryza sativa* Japonica Group (Os) and *Physcomitrella patens* (Pp) were used for the alignment. ClustalW multiple alignments were visualized using BioEdit ver. 7.2.5 and colored by percentage identity (black) and similarity (gray).

A

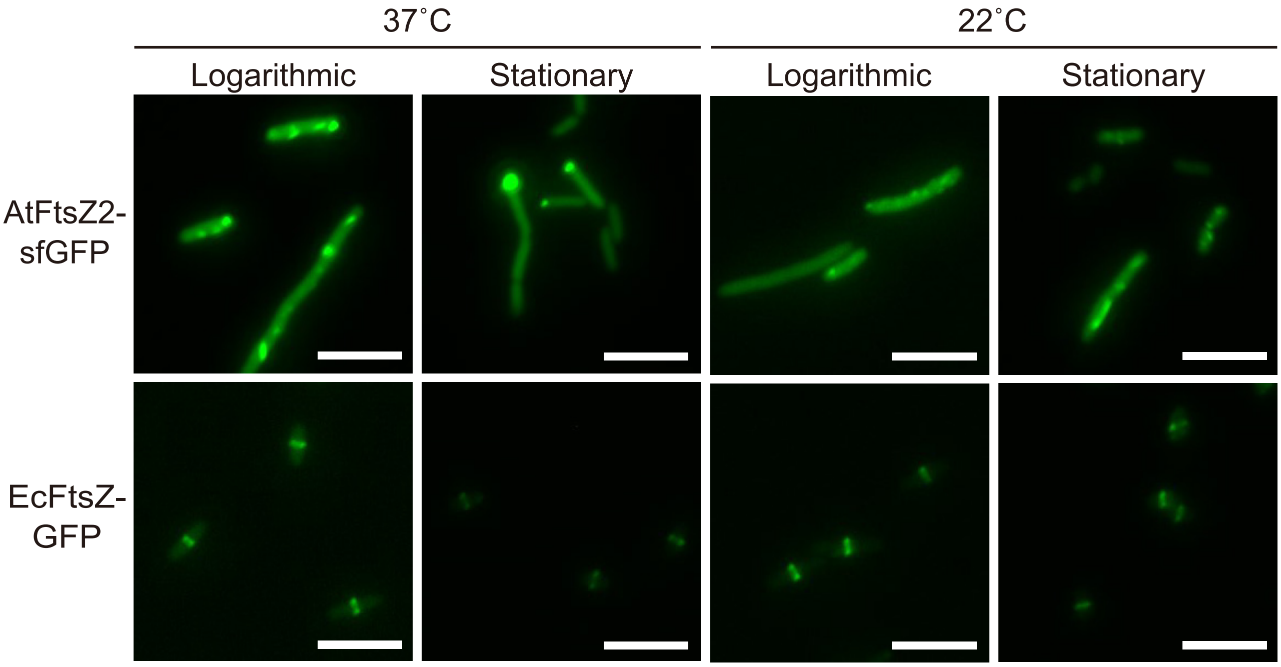

B

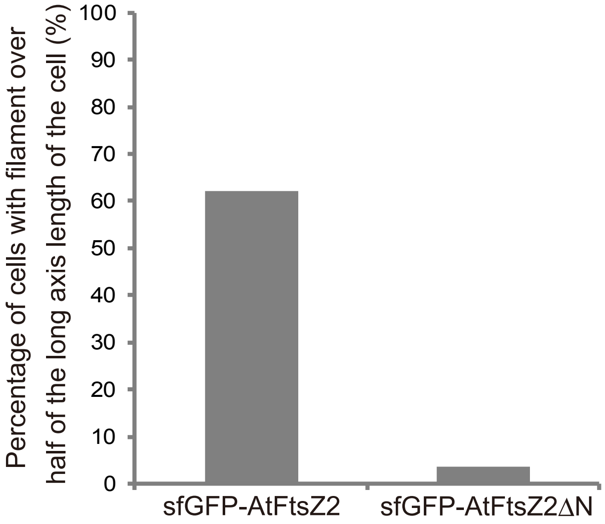

Figure S2. Localization pattern of C-terminally fluorescent protein-fused AtFtsZ2 and quantification of AtFtsZ2 filaments with or without its N-terminus.

(A) Fluorescent images of AtFtsZ2-sfGFP and EcFtsZ-GFP at various conditions. Bars=5μm.

(B) Quantification of the effect of AtFtsZ2 N-terminal region on filamentation. The graph shows the percentage of cells containing sfGFP-AtFtsZ2 filaments that extended over half the cell length along the long axis. At least 100 bacterial cells were investigated for the quantification.

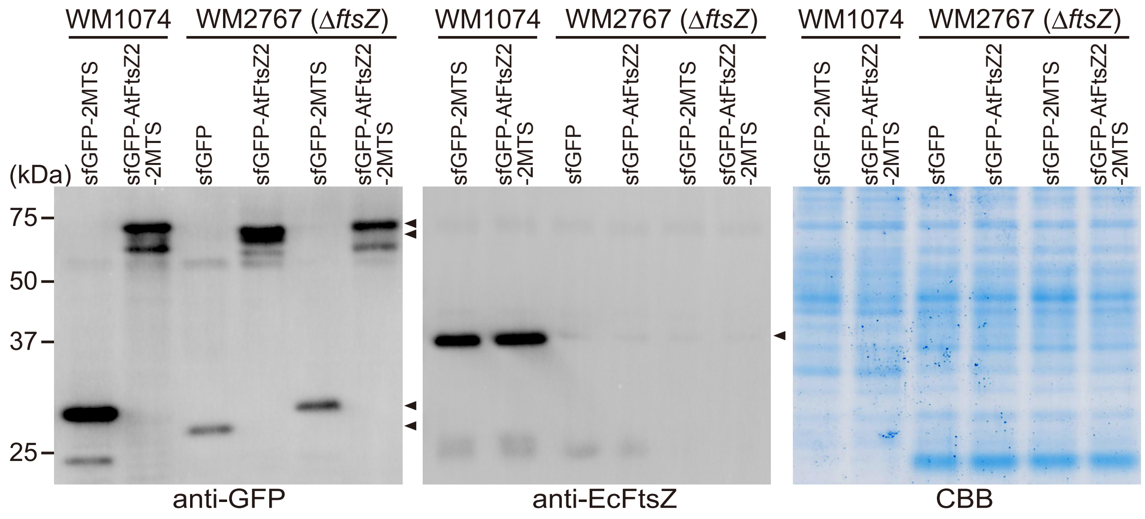

Figure S3. Immunoblot analysis of FtsZ homologues at various conditions.

sfGFP-fused proteins and endogenous EcFtsZ were detected by anti-GFP and anti-EcFtsZ antibodies, respectively. Arrowheads indicate full-length band of each protein. Coomassie staining was performed as a loading control.

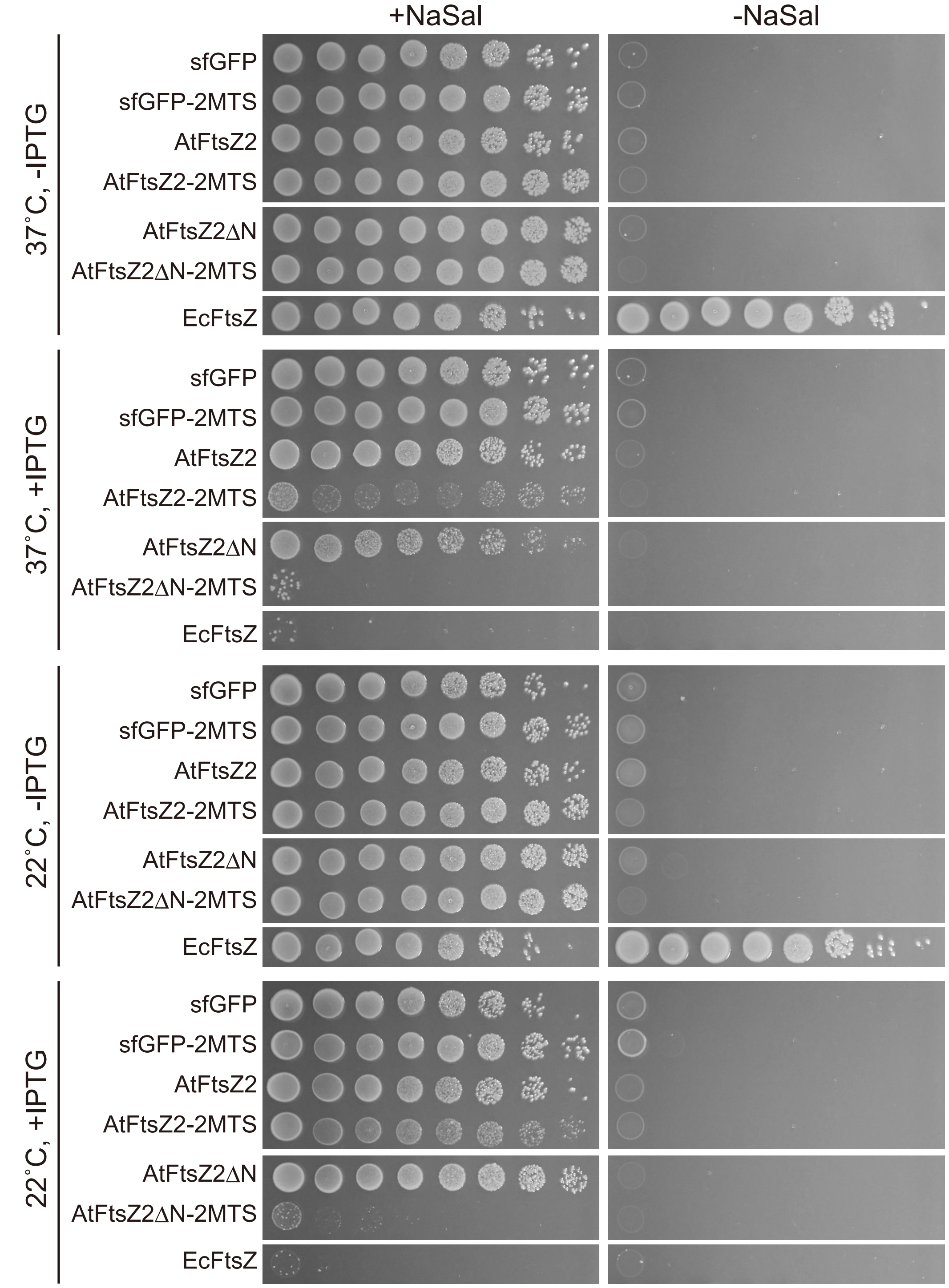

Figure S4. Complementation assay of various AtFtsZ2 constructs in FtsZ-depleted strain WM2767. Depletion of endogenous EcFtsZ was achieved by removal of NaSal in bacterial culture. The expression of AtFtsZ2 constructs and EcFtsZ is induced by IPTG. The plates were incubated for 22 h (37˚C) and 77 h (22˚C), respectively.

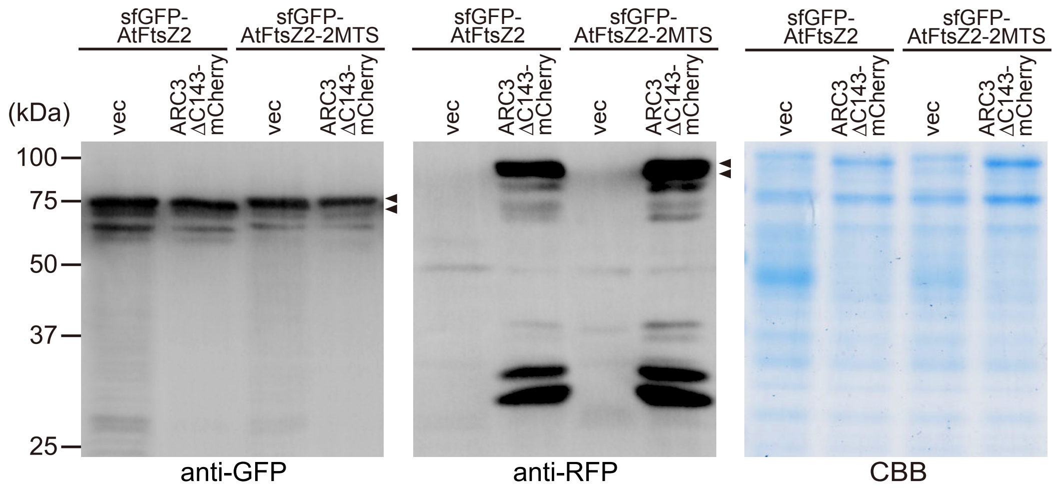

Figure S5. Immunoblot analysis of FtsZ homologues and/or ARC3.

sfGFP-fused AtFtsZ2 and mCherry-fused ARC3 expressed in WM1074 cells were detected by anti-GFP and anti-RFP antibodies, respectively. Arrowheads indicate full-length band of each protein. Coomassie staining was performed as a loading control.

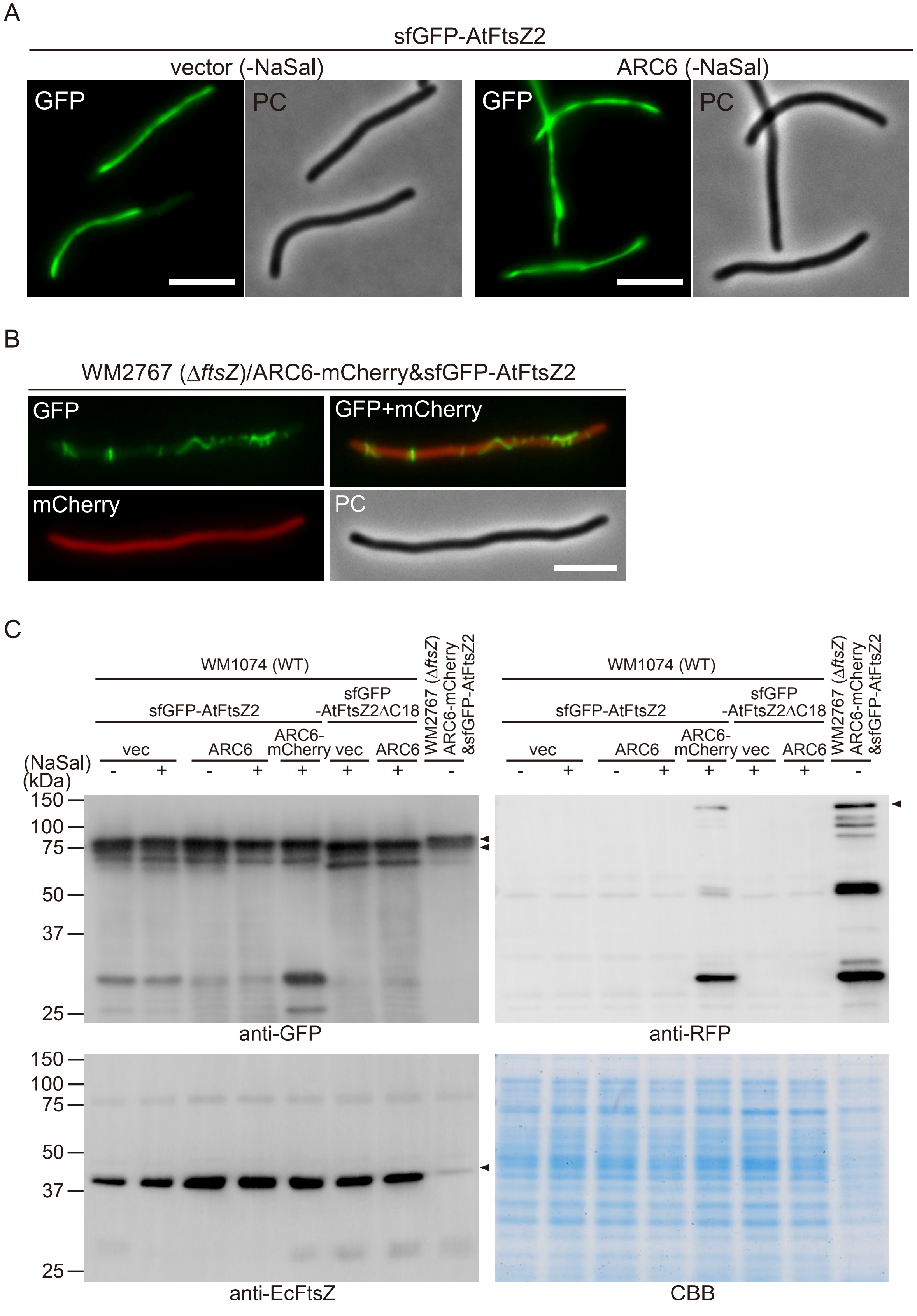

Figure S6. The effects of ARC6 on filament morphology of sfGFP-AtFtsZ2 and immunoblots of FtsZ homologues and/or ARC6.

(A) Fluorescent image of sfGFP-AtFtsZ2 in WM1074 strain in the absense of NaSal. Bars=5μm.

(B) Concurrent observation of sfGFP-AtFtsZ2 Z ring-like structures and ARC6-mCherry in WM2767 strain. Bar=5μm.

(C) sfGFP- and mCherry-fused proteins and endogenous EcFtsZ were detected by anti-GFP, anti-RFP and anti-EcFtsZ antibodies, respectively. Arrowheads indicate full-length band of each protein. Coomassie staining was performed as a loading control.

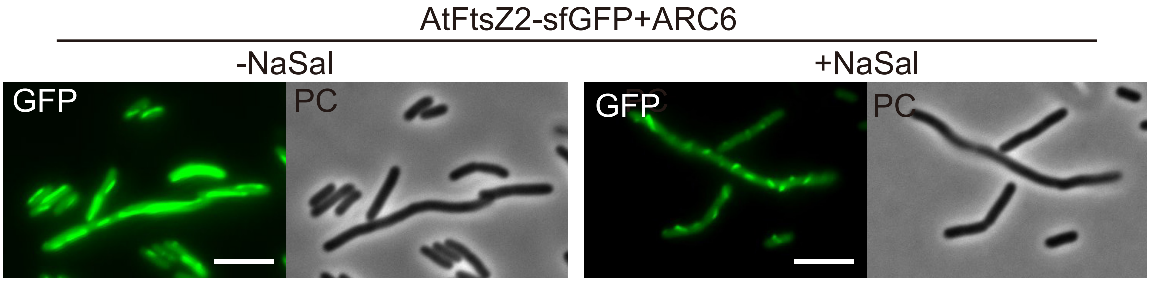

Figure S7. The effects of ARC6 on localization pattern of C-terminally fluorescent protein-fused AtFtsZ2. Filament morphology of AtFtsZ2-sfGFP with or without NaSal (an inducer for expression of ARC6) in WM1074 strain at 22˚C. Bars=5μm.

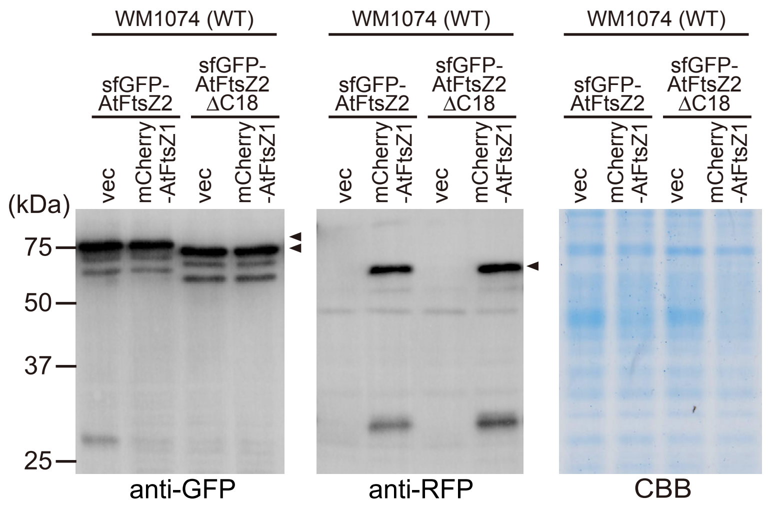

Figure S8. Immunoblots of AtFtsZ1 and AtFtsZ2.

sfGFP-AtFtsZ2 and mCherry-AtFtsZ1 were detected by anti-GFP and anti-RFP antibodies, respectively. Arrowheads indicate full-length band of each protein. Coomassie staining was performed as a loading control.
